# Alterations Functional Connectivity in Temporal Lobe Epilepsy and Their Relationships With Cognitive Function: A Longitudinal Resting-State fMRI Study

**DOI:** 10.3389/fneur.2020.00625

**Published:** 2020-07-21

**Authors:** Lu Qin, Wenyu Jiang, Jinou Zheng, Xia Zhou, Zhao Zhang, Jinping Liu

**Affiliations:** ^1^Department of Neurology, Liuzhou Workers’ Hospital/The Fourth Affiliated Hospital of Guangxi Medical University, Liuzhou, China; ^2^Department of Neurology, Jiangbin Hospital of Guangxi Zhuang Autonomous Region, Nanning, China; ^3^Department of Neurology, The First Affiliated Hospital of Guangxi Medical University, Nanning, China

**Keywords:** temporal lobe epilepsy, functional magnetic resonance imaging, dorsolateral prefrontal cortex, longitudinal study, cognitive function

## Abstract

**Background:** Cognitive impairments in temporal lobe epilepsy (TLE) patients has been described as a chronically progressive feature of the disease. However, how severe recurrent seizures modify neuronal circuits in the human brain and subsequently degrade cognitive function, remains largely unknown. Here, we aimed to investigate longitudinal alterations by functional magnetic resonance imaging (fMRI) in TLE patients and to assess how those alterations are related to cognitive function performance.

**Methods:** Sixteen TLE patients and 20 normal controls (NCs) were recruited for a study to observe longitudinal alterations in resting-state functional connectivity (FC) and to estimate alertness, orientation, and executive function both at baseline and at a follow-up time ~3 years later.

**Results:** TLE patients, compared with NCs, showed impaired executive function, intrinsic alertness, and phasic alertness and exhibited lengthened reaction time (RT) in the spatial cue and center cue conditions at baseline. The orienting function of TLE patients was declined at follow-up compared to the baseline. Cross-sectional analysis demonstrated that TLE patients displayed significantly greater positive correlation than NCs between the right dorsolateral prefrontal cortex (DLPFC) and the right inferior parietal lobule (IPL) and right superior frontal gyrus (SFG). Furthermore, among TLE patients, the longitudinal study revealed a decrease in correlation between the right DLPFC and the right SFG compared to the baseline. In addition, there was a significant negative correlation between the longitudinal change in FC and the change in orienting function in TLE subjects.

**Conclusions:** Abnormal connectivity between the DLPFC and the SFG suggests the potential of longitudinal resting-state fMRI to delineate regions relevant to cognitive dysfunction for disease progression.

## Introduction

Temporal lobe epilepsy (TLE), a chronic disorder characterized by severe recurrent seizures, frequently occurs in patients with epilepsy and is associated with decreased cognitive ability in an extensive range of domains, although most seizures can be controlled by anti-epileptic drugs (1). Prominent deficits that fall under the general term “cognitive impairment” have been detected in a cluster of abilities, such as attention, memory, and language, and may continuously deteriorate as epilepsy progresses (2). Previous studies have shown that memory deficits are observed in most epilepsy patients (3). However, cognitive impairment is easily overlooked. Often, by the time cognitive decline is detected, the brain damage has spread considerably (4) due to lack of timely treatment. Therefore, it is particularly important to seek relevant indicators of early-stage cognitive impairment in TLE.

Given the importance of cognitive function for independent living and social function, cognitive impairment has been proposed as a likely contributor to the wide range of dysfunction that emerges in TLE (5). Cognitive impairment is of central interest and is correlated with patient functional outcomes. Attention precedes cognition (6). The importance of attention is its unique role in preparing and sustaining high sensitivity to process high-level cognitive tasks. Meanwhile, attentional function plays an indispensable role in all aspects of human behavior (7). As proposed by Posner (8), the attention network involves the alertness network, the orientation network, and the executive control network. Alertness, a fundamental form of attention, is the ability to maintain and improve reaction sense to incoming stimuli. Alertness consists of two components: intrinsic alertness and phasic alertness. Intrinsic alertness refers to the ability to sustain internal arousal in the absence of any external stimuli. Phasic alertness refers to the ability to enhance response readiness in reaction to an external warning stimulus (9). Orientation is managed by showing a central cue or a spatial cue showing where in space a person should focus; the movement of the eyes to the target offers a foundation for attention to the cued position. In addition, executive function, or cognitive control, assists with planning, reasoning, and problem solving, allows one to hold information in working memory, to inhibit highly automated responses to stimuli, and to shift the focus of attention between related but distinct aspects of a given task or problem (10, 11).

To date, the attention network test (ANT), proposed by Fan et al. (12), has been extensively used to estimate the efficiency of three components of the attention network. Previous studies have verified the utility of the ANT in evaluating several modulators of attentional function. In addition, our studies have used the test to identify deficits in alertness and executive function (13, 14). Based on neuropsychology, neuroanatomy, and neuroimaging, Posner (8) proposed the attention network theory, in which the attention network is divided into alertness, orientation, and executive control components. According to this theory, Fan et al. designed a brief test, known as the attentional network test (ANT), by combining classic cued tasks with the flanker task. The ANT can evaluate alertness by changing the cues and can accurately describe the alertness, orientation, and executive function of the attention network (15). The ANT has widely been used in neurocognitive and neuroimaging research and in research on alertness in patients with depression, attention-deficit/hyperactivity disorder (ADHD), and Alzheimer's disease (AD) (16).

By virtue of its advantage in comprehensively analyzing the morphology and function of the regions of interest with different weighted modes, multimodal MRI has become the main method used for the non-invasive neurophysiological study of the human brain. A systematic review of cognitive function in patients with TLE revealed that structural brain abnormalities including gray matter pathology and white matter reductions are related to cognitive dysfunction (17). This finding suggests that structural abnormalities determine cognitive deficits and may be a common pathophysiology of cognitive degeneration and the occurrence and progression of epileptic seizures. Some anatomical structures such as the dorsolateral prefrontal cortex (DLPFC) may therefore account for cognitive dysfunction in TLE (18, 19).

As described above, attention function, including alertness, orientation, and executive control function, consistently deteriorate with disease progression in TLE patients, resulting in clinically significant cognitive decline. Longitudinal alterations in DLPFC resting-state functional connectivity may result in abnormalities manifested as disease aggravation. Since functional connectivity (FC) is defined as the spatial-domain correlation between spatially distant brain regions, altered FC among brain regions can reflect brain reorganization and abnormal functional interactions (20–22). We therefore planned a 3 year longitudinal study, aiming to estimate alertness, orientation and executive control network efficiency to determine whether cognitive deficiency exists in TLE patients compared to normal controls (NCs), as well as whether cognitive deficits increase longitudinally, to clarify whether longitudinal alterations in DLPFC resting-state FC can be observed in TLE patients at follow-up, and finally, to identify correlates of cognitive performance.

## Materials and Methods

### Patients

Sixteen TLE patients [age =27.53 ± 6.63 years, education = 11.47 ± 3.27 years, eight males, eight females] who attended the Epilepsy Clinic of the First Affiliated Hospital of Guangxi Medical University were recruited for the epilepsy group. According to the criteria of the International League Against Epilepsy (ILAE), all of the TLE patients satisfied at least two of the following inclusion criteria (23): (1) typical symptoms of seizures, involving epigastric rising, abnormal emotional experiences and psychiatric symptoms, automatisms, and dystonic posturing of the limbs, suggesting that the epileptogenic focus was located in the temporal lobe; (2) MRI revealing hippocampal sclerosis, atrophy, or aberrant signal in the temporal lobe; (3) an ictal or interictal electroencephalogram suggesting epileptic discharges in the temporal lobe; and (4) receiving relevant anti-epileptic drug treatment, and adjustment of the medicine plan in the Epilepsy Clinic of the First Affiliated Hospital of Guangxi Medical University according the patient's clinical symptoms. In addition, TLE patients were eliminated if they simultaneously suffered from psychiatric diseases, serious diseases in other systems, or any neurological disorders other than TLE.

Twenty NCs matched to the overall patient group by age, gender, and mean years of education (mean age ± SD = 27.19 ± 3.82 years, mean years of education ± SD = 12.31 ± 2.89 years, 12 males) were recruited. They were excluded if they had a history of epilepsy or any other chronic neurological or psychiatric disease.

The project was administered by the Ethics Committee of the First Affiliated Hospital of Guangxi Medical University, Nanning, China. All of the subjects were required to provide signed informed consent in order to participate and were instructed in detail about the experiment.

### Neuropsychological Assessment of Cognitive

Alertness, orientation and executive performance were assessed using the ANT proposed by Fan et al. (12), which is a generally accepted neuropsychological assessment established on the E-prime platform. All of the participants were instructed to concentrate on the test. Each cue consisted of a line of five arrows, each pointing either left or right. The distance from participants' eyes to the screen was about 60 cm. All participants completed three blocks (alerting block, orienting block, and executive control block) of the ANT. The order of the three blocks was counterbalanced across subjects. Each block took 424 s, which contained a buffer time of 4 s, two practice trials of 10 s, and 40 experimental trials of 10 s. Each trial began with a fixation or cue for 100 ms which was followed by 300 ms fixation. After that, a target (congruent or incongruent, central, or spatial) appeared for 2,000 ms or until the participant pressed a key. Last, another fixation was presented to ensure the overall time of one trial was 10,000 ms (0.1 Hz). The participant was required to rapidly identify the direction of the middle arrow and to indicate it by pressing a button. Additionally, double asterisks, a central asterisk, an upper or lower asterisk, or no asterisk was presented during each trial. Before the formal experiment, 24 practice trials were delivered to acquaint subjects with the procedure and operating instructions and to improve accuracy. Attentional function includes three basic components: alertness, orientation and executive control. Alertness, orientation, and executive function were defined quantitatively by the following formula: mean RT_no−cue_–mean RT_double−cue_, mean RT_center−cue_–mean RT_spatial−cue_, RT_incongruent_–RT _congruent_ (24).

### Data Acquisition

The participants were scanned using an Achieva 3T MRI scanner (Philips, the Netherlands) with a 12-channel head coil. Resting-state fMRI scans were collected for a gradient-echo echo planar imaging (EPI) sequence: repetition time = 2,000 ms, echo time = 30 ms, number of slices = 31, slice thickness/gap = 5/1 mm, acquisition matrix = 64 × 64, field of view = 220 mm × 220 mm, flip angle = 90°, and 180 dynamics. The participants were required to stay still, keep their eyes closed, avoid thinking about anything other than the task, and remain awake. In order to reduce noise and prevent head motion, headphones and padding were used throughout the scans. The entire acquisition lasted ~6 min.

### Data Processing

The resting-state fMRI data were processed using DPABI (http://resting-fmri.sourceforge.net) software, which is based on the MATLAB R2013b platform. First, the first 10 images were excluded to guarantee the stability of the data. Slice timing and realignment were applied to each participant, and any subject with head motion of more than 2.5 mm of translation or 2.5°of rotation was removed from the analysis. In our study, no subjects were discarded according to these criteria. Second, the images were normalized to a conventional EPI template in the Montreal Neurological Institute (MNI) space and resampled to 3 mm × 3 mm × 3 mm. Subsequently, smoothing (6 mm full width at half-maximum Gaussian kernel) and bandpass filtering (0.01 to 0.08 Hz) were performed to reduce linear trends and lessen the impacts of high-frequency noise and low-frequency drift. Finally, several nuisance covariates, including the six head motion parameters, the global mean signal, the white matter signal, and the cerebrospinal fluid signal, were regressed out (25, 26).

### Functional Connectivity Analysis

The WFU PickAtlas toolkit, which is based on statistical parametric mapping, was utilized to extract the two regions of interest, or ROIs (right DLPFC and left DLPFC), in preparation for FC analysis (27, 28). In our study, the bilateral DLPFC was defined as the portion of the bilateral middle frontal gyrus within Brodmann area 46 (BA46).

The RESTplus toolbox was used for the subsequent seed-based whole-brain-related FC analysis. The mean time course of the left DLPFC and right DLPFC in each individual subject was extracted to perform Pearson's correlation analysis between each ROI and all other brain voxels following the generated correlation maps. In order to improve the normality of the correlation coefficients, Fisher's r-to-z transformation was employed to generate zFC images.

### Longitudinal Study

Each of the TLE patients underwent a follow-up ANT and fMRI scan after 3 years (31.3 ± 9.3 months). There was no obvious alteration in cognitive function between the TLE patients and the NCs, who were selected among healthy adults. The 20 NCs were tested only once at baseline; the process was otherwise the same for them.

### Statistical Analysis

Demographic data and ANT results were analyzed utilizing SPSS16 software. Continuous variables, including age, years of education, and ANT results, were compared by two-sample *t*-tests, while the difference in gender composition between the TLE and control groups at baseline was assessed using the chi-squared test, and paired-sample *t*-tests (*p* < 0.05) were conducted for longitudinal comparisons between baseline and follow-up in TLE patients.

RESTplus software was applied for statistical analyses of the zFC data. First, we performed one-sample *t*-tests to determine the brain regions that were positively correlated with the left or right DLPFC. A mask was then created by combining the significant areas on the zFC maps of the TLE and NC groups according to the one-sample *t*-tests. Subsequently, two-tailed two-sample *t*-tests were conducted to search for substantial differences in DLPFC FC between TLE groups and controls at baseline. Finally, two-tailed paired-sample *t*-tests were applied for the longitudinal analysis to determine the significance of the difference in the DLPFC FC between baseline and follow-up in the TLE patients; all these analyses used a statistical significance level of *p* < 0.05 (GRF corrected, cluster *P* < 0.001, voxel *P* < 0.05).

Significant FC alterations in the DLPFC of TLE patients between baseline and follow-up were defined in terms of ROIs, and the individual mean time courses of the regions were extracted. Pearson's correlation analysis was then performed to explore the relationships between alterations in DLPFC FC and changes in alerting, orienting, and executive performance in TLE patients.

## Results

### Demographic Characteristics

No significant difference was found in age, gender, or educational level between the TLE patients and NCs (Table 1).

**Table 1:** Demographic characteristics of TLE patients and NCs.

	**Age** **(years)**	**Gender** **(male/** **female)**	**Education** **(years)**	**Handedness** **(right/left)**	**Duration of epilepsy (years)**
TLE	29.6 ± 7.0	8/8	14.2 ± 3.6	16/0	10.8 ± 8.3
NCs	26.3 ± 5.6	12/8	15.3 ± 3.7	20/0	/
*P*-value	1.591	0.360	−0.863	/	/

*Two-sample t-tests were compared with the two groups, except for “gender” where the p-value was obtained using the χ2-test*.

### ANT Results

**Figure 1:**
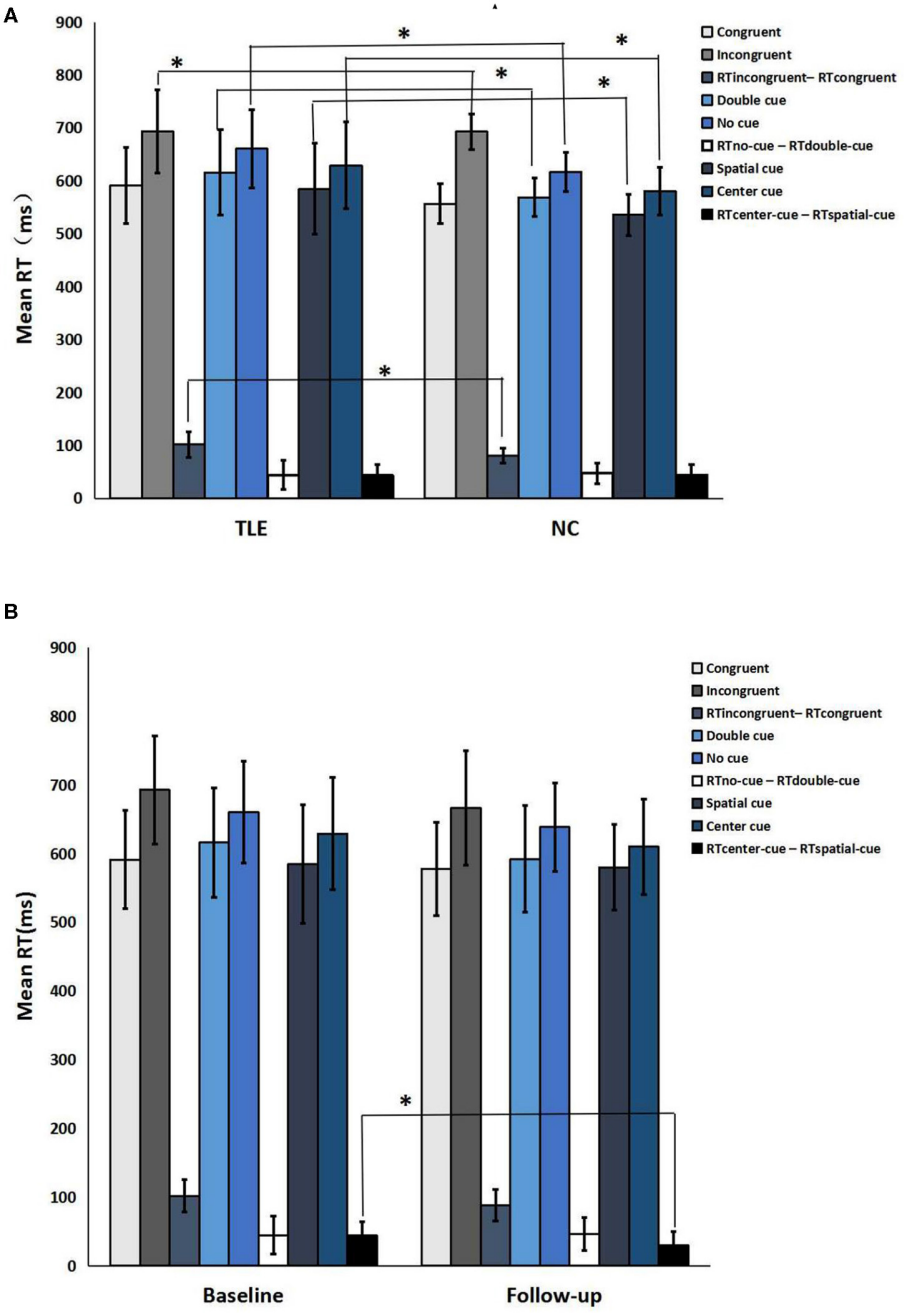
Neuropsychological attention network test performance. **(A)** TLE patients vs. normal controls; **(B)** Follow-up vs. baseline. *a significant difference in the two groups (*p* < 0.05).

Compared with the controls, TLE patients showed significantly increased intrinsic alertness, phasic alertness, spatial cue RT, center cue RT, and executive function at baseline. However, there was no significant difference in the effectiveness of alertness or orienting in TLE patients (Table 2 and Figure 1A). Orienting function declined over time in the TLE patients (Table 3 and Figure 1B).

**Table 2:** Neuropsychological attention network test performance of TLE patients and NCs.

	**Congruent (ms)**	**Incongruent (ms)**	**RT_**incongruent**_–RT_**congruent**_ (ms)**	**Double cue (ms)**	**No cue (ms)**	**RT_**no-cue**_–Rt_**double-cue**_ (ms)**	**Spatial cue (ms)**	**Center cue(ms)**	**RT_**center-cue**_–RT_**spatial−cue**_ (ms)**
TLE	591.4 ± 71.8	693.2 ± 78.6	101.8 ± 23.8	616.2 ± 80.1	660.7 ± 74.2	44.5 ± 27.4	585.0 ± 86.1	629.1 ± 81.9	44.0 ± 20.0
NCs	556.8 ± 37.3	636.3 ± 33.4	80.4 ± 14.2	569.1 ± 35.7	616.8 ± 36.8	47.6 ± 19.2	536.0 ± 38.7	580.8 ± 45.4	44.9 ± 19.5
*P*-value	0.070	0.006^*^	0.002^*^	0.024^*^	0.026^*^	0.689	0.029^*^	0.032^*^	0.902

* *P < 0.05 represents significant decline in the two groups*.

**Table 3:** Neuropsychological attention network test performance of TLE patients between baseline and follow-up.

	**Congruent (ms)**	**Incongruent (ms)**	**RT_**incongruent**_–RT_**congruent**_ (ms)**	**Double cue (ms)**	**No cue (ms)**	**RT_**no-cue**_–Rt_**double-cue**_ (ms)**	**Spatial cue (ms)**	**Center cue (ms)**	**RT_**center-cue**_–RT_**spatial-cue**_ (ms)**
Baseline	591.4 ± 71.8	693.2 ± 78.6	101.8 ± 23.8	616.2 ± 80.1	660.7 ± 74.2	44.5 ± 27.4	585.0 ± 86.1	629.1 ± 81.9	44.0 ± 20.0
Follow-up	578.1 ± 68.2	666.6 ± 83.0	88.5 ± 23.0	592.5 ± 77.9	638.7 ± 64.8	46.2 ± 24.2	580.1 ± 62.2	610.4 ± 69.5	29.9 ± 19.9
*P*-value	0.241	0.050	0.057	0.180	0.160	0.787	0.824	0.335	0.032^*^

* *P < 0.05 represents significant decline in the two groups*.

### Functional Connectivity Result

#### Cross Sectional Analyses Between TLE Patients and Controls

**Figure 2:**
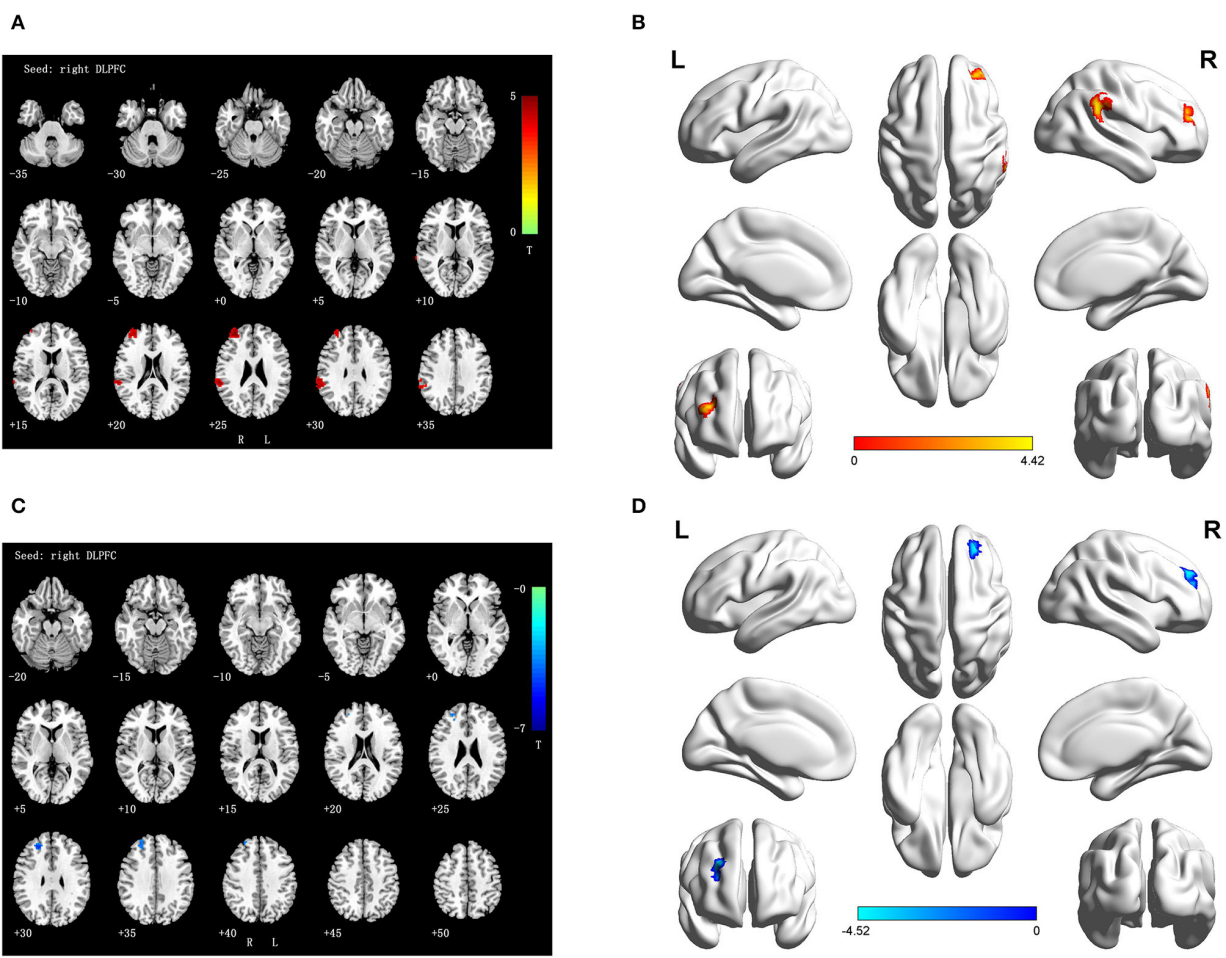
**(A,B)** Differences FC of right DLPFC in TLE subjects as compared to NCs at baseline (interaction effect); **(C,D)** Differences FC of right DLPFC in TLE subjects at follow-up vs. baseline (interaction effect). Numbers in the figure indicate the Z coordinate in MNI space; FC, functional connectivity; DLPFC, the dorsolateral prefrontal cortex; R, right; L, right; Warm color represent positive correlation brain region in TLE patients at follow-up as compared with baseline; cool color represent negative correlation brain region in TLE patients at follow-up as compared with baseline.

Cross-sectional analyses of fMRI showed positive correlation between TLE patients and NCs in brain regions associated with the right DLPFC, namely, the right inferior parietal lobule (IPL) and the right superior frontal gyrus (SFG) (Table 3 and Figures 2A,B).

#### Longitudinal Analyses Result Between Follow-Up and Baseline

During the longitudinal study, compared with the baseline, a negative activity correlation with the right DLPFC appeared in the right SFG of the TLE patients (Table 4 and Figures 2C,D).

**Table 4:** Brain regions showed significantly difference in the two groups.

**Region**	**MNI coordinates**			**Cluster size (voxel)**	***T*-value**
	**X**	**Y**	**Z**		
**TLE vs. NCs**					
**Right DLPFC**					
right inferior parietal lobule (BA40)	60	−39	27	139	4.3239
right superior frontal gyrus (BA10)	33	51	24	98	4.4227
**Follow-up vs. baseline**					
**Right DLPFC**					
right superior frontal gyrus (BA10)	24	39	30	57	−4.5222

MNI, Montreal Neurological Institute; DLPFC, the dorsolateral prefrontal cortex.

T > 0 represent positive correlation brain region in TLE patients as compared with NCs.

*T < 0 represent negative correlation brain region in follow-up of TLE patients as compared with baseline. Between-group results were a multiple comparisons using GRF theory*.

### Correlations Analysis Results

**Figure 3:**
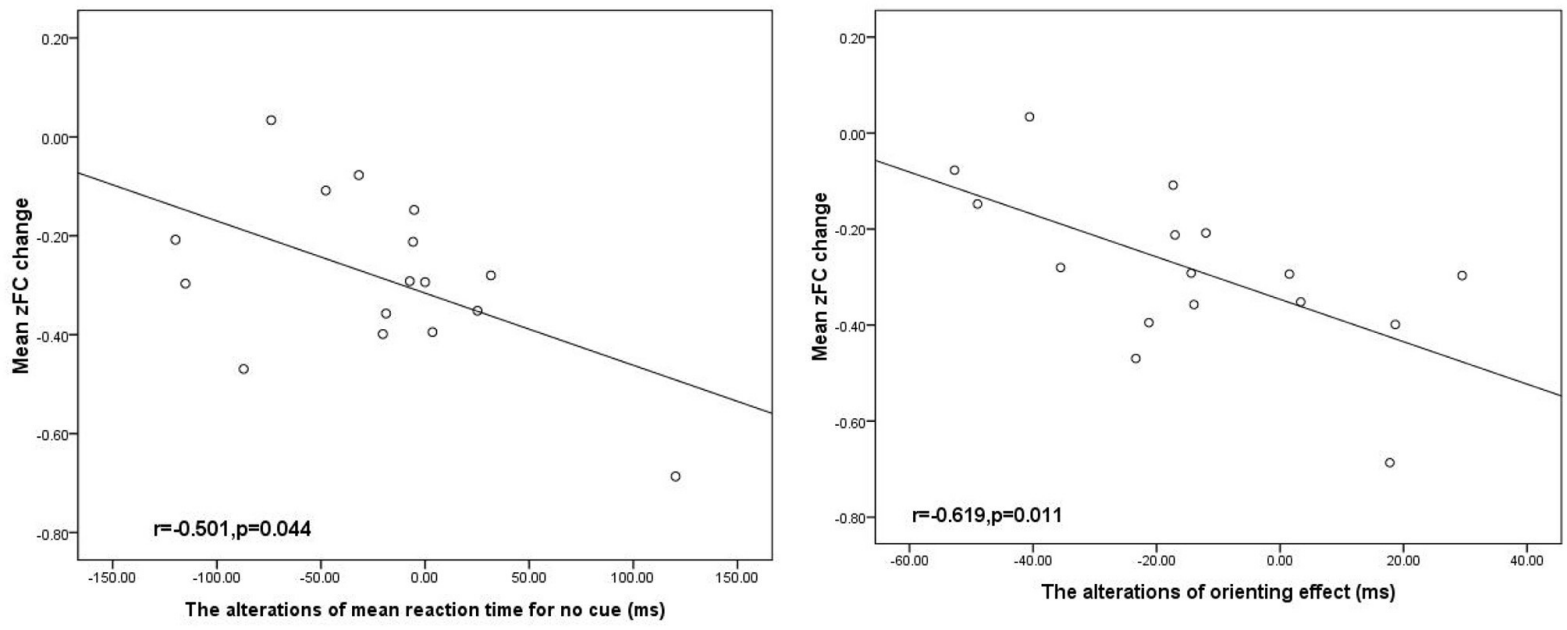
Negative significant correlation between the alteration DLPFC FC associated with the SFG and the orienting effect change in the patients with TLE (*p* < 0.05). SFG, superior frontal gyrus; DLPFC. R, right dorsolateral prefrontal cortex, and ms, millisecond.

The average FC values extracted in ROIs derived from longitudinal alterations in FC in the DLPFC between baseline and follow-up were significantly negatively correlated with the changes in orienting effect in TLE subjects (Figure 3).

## Discussion

To our knowledge, this was the first study to track TLE patients over a 3 year period using advanced acquisition fMRI to investigate longitudinal alterations associated with cognitive performance. Since TLE is a chronic progressive disease, cognitive deficits often accelerate as the disease progresses, although no obvious change occurs at the time of initial epilepsy diagnosis. It is necessary to track the alterations in TLE patients by combining fMRI with neuropsychological testing using the ANT. Regarding the ANT results, we found that TLE patients showed impaired alertness, orientation, and executive function compared with NCs and demonstrated declined orientation function at follow-up compared with the baseline. For the fMRI analysis, we determined that TLE patients displayed significantly positive correlation differences between the right DLPFC and the right IPL and SFG at baseline, and a longitudinal study exhibited negative correlation differences in the right DLPFC with right SFG at follow-up. In addition, there was a significant negative correlation between longitudinal changes in FC and alterations in the orienting effect in TLE subjects.

Attention function, the basis of cognitive function, refers to alertness, orientation, and executive performance, which are necessary to independently carry out tasks involving working memory, planning, organization, and problem-solving strategies (29). In our study, we found that intrinsic alertness, phasic alertness, mean RT for spatial cues, mean RT for center cues, and executive function were significantly increased when there was no difference in alertness and orientation effect in TLE patients relative to NCs at baseline. In general, these data reveal a potential cognitive decline in TLE patients, which was consistent with several other studies conducted by Schraegle and Nussbaum (30), Li et al. (31), Nau et al. (32), and van den Berg et al. (33). TLE is associated with decreases in cognitive function, such as attention, memory and language, which may be present in most patients with epilepsy (34). A literature review by Cettina that assessed the neuroimaging signs of TLE and cognitive dysfunction also demonstrated that significant cognitive decline, particularly in memory, alertness and executive function, was observed in patients with TLE (35).

We initially hypothesized that patients would present a magnified decrease in functional connectivity in some related areas over time, concomitant with worse cognitive performance. Contrary to our hypothesis, TLE patients, compared with the baseline, exhibited slight improvements in cognitive performance over 3 years, although the differences were not significant except for orientation function. More regular usage of anti-epileptic drugs, better control of seizures, and shorter disease duration in people with TLE may contribute to improved cognitive function. This may explain why the cognitive function of our patients displayed no obvious change during the 3 years. In agreement with other results, a study of TLE patients over a 13 year period also showed no significant change in cognitive function (36). In addition, Helmstaedter and Elger (37) and Krámská et al. (38) revealed, based on their longitudinal-sectional study of declarative cognitive functioning in chronic refractory partial epilepsy, that there was no deterioration in neuropsychological performance after a long period of epilepsy. The ANT scores are remarkably stable, with only a decline the orientation effect over time in our results. The reason may be that orientation effect is more sensitive than other cognitive function. More longitudinal assessments remain to be investigated in future studies. Nevertheless, Hermann et al. (39) discovered that in TLE patients, cognitive function deteriorated considerably by 15–20% over 4 years, which was attributed to aging, seizure frequency, and anatomical abnormalities on MRI. Low intelligence and long disease duration have been linked to adverse cognitive outcomes. Our research excludes other cognitive functions, such as language and memory, which remain to be studied in depth experimentally.

Concerning fMRI analysis, we selected the DLPFC as the ROI. The DLPFC, a key cortical region, is widely accepted to be substantially involved in cognitive function. According to a study by Haber et al. (40), one of the pathways of the frontostriatal circuits is a dorsal control pathway (DLPFC projecting to caudate) involved in cognitive control (achieving future goals and inhibiting the prepotent response). Quantitative MRI studies have confirmed that decreased dorsal prefrontal cortex volume is significantly correlated with cognitive impairment (41). The hypothesis of a central role in cognitive deficits is further supported by findings that TLE patients display gray matter reductions and structural abnormalities in DLPFC areas that support cognitive function, in addition to a decrease in abnormalities in temporal brain regions (42). While the features of aberrant DLPFC structure have been described, the mechanism by which TLE pathology relates to cognitive impairment has not been well-established. Studies have shown that volume atrophy of the DLPFC may account for deficits in cognitive functioning (43, 44). Previous analyses demonstrated that decreases in gray matter volume and cortical thickness frequently occurred in TLE patients (45). Tan et al. (46) revealed significant metabolic changes in TLE patients, and the reductions in the left DLPFC were greater in patients than in controls. This result implies that TLE can lead to metabolic changes in the DLPFC, which is distant from the seizure focus. Furthermore, reduced DLPFC correlation during affective tasks was observed in schizophrenia patients; this region might be a core region of the cognitive control network (47). Alterations of the FC in the DLPFC were significantly linked to cognitive function and appeared to be a reliable predictor of impaired cognitive function in TLE subjects within a relatively short period of 3 years. Here, we investigated the considerably decreased FC of the DLPFC over a 3 year follow-up period in TLE subjects. The current results suggest a crucial contribution of DLPFC dysfunction to cognitive impairment in TLE.

As for the results of cross-sectional analysis, the differences between TLE patients and NCs were obvious at the baseline assessment. TLE patients showed increased parietal (IPL) and frontal (SFG) activation compared to controls. Considering the increased activation in these regions, we speculated the existence of a strong compensatory mechanism associated with decreased cognitive performance. The IPL, a region that constitutes part of the default mode network, contributed to cognitive impairment in TLE patients (48). Considering the significantly reduced cognitive function as demonstrated by the ANT, it is reasonable to speculate that there was an abnormally increased level of DLPFC FC, acting as a compensatory mechanism for cognitive functional deficits. Our findings are consistent with previous studies, which found increased correlation in the frontal and parietal lobes of TLE patients (13, 14), indicating that these regions play a crucial role in cognitive control (49). In line with a prior study (50), increased activation was found in certain frontoparietal regions of TLE patients. The authors of the previous work suggested that the increase in SFG and IPL activation reflects resource recruitment to compensate for cognitive dysfunction in TLE patients, which might also be the case in our study. Intriguingly, no negative activation difference in any brain region was observed between the groups. However, the specific mechanism remains obscure. In addition, we cannot ignore the implications of the relatively rigorous significance level we chose for reporting our results. Our result was incompatible with those in the literature that found significant negative activation differences in the brain (51). The exact mechanism of the abnormal DLPFC FC related to cognitive dysfunction in TLE patients remains to be further explored.

Consistent with our expectations, in the longitudinal assessment, TLE patients demonstrated significantly decreased FC between the right DLPFC and right SFG compared with the baseline, while the region exhibited a positive activation difference compared with the controls at the baseline analysis. It is not surprising that these regions showed aberrant FC, given that the SFG is part of the frontal lobe, which is associated with cognitive function (52). In studies related to cognitive function, it has been suggested that there was temporarily increased activation of FC in the frontal region to compensate for cognitive dysfunction over a period of time, followed by a slow, significant reduction from which the brain does not recover (53). Therefore, our results confirm findings from earlier studies showing that the FC of the SFG in TLE patients is decreased and constantly deteriorates over time. Our findings are also in concordance with prior longitudinal studies by Tang et al. (54), who collected rs-fMRI data ~4.5 months after surgery in patients with unilateral mesial TLE, and from Hafkemeijer et al. (55), who collected rs-fMRI data over approximately 1.8 years in patients with AD, both indicating that the region plays a crucial role in cognitive function. Furthermore, previous studies reported that the SFG was one of the key brain regions involved in cognitive function in TLE and AD patients (56) and that aberrant FC between the SFG and other brain regions contributed to cognitive impairment. In agreement with the aforementioned studies, a reduction in the FC between the DLPFC and the SFG may highlight the role of the frontal lobe in the cognitive function of TLE and progression of TLE.

In general, regardless of rather stable performance in the ANT, this finding supports the notion that TLE patients suffer considerable ongoing damage due to ictal and interictal epileptic discharge, reflected in the observation that the patients who had no regions with significantly decreased activity at baseline developed considerable decreases over time. However, we cannot exclude the interference of clinical parameters, including age of onset, duration of epilepsy, seizure type, and utilization of anti-epileptic drugs. Mood changes can also influence results—both clinical and experimental studies (57, 58) have shown that depression and anxiety are common in TLE, triggering further brain damage that can deteriorate the epileptic condition and lead to accelerated cognitive impairment. In addition, it is worth noting that the decreased activity of the DLPFC in patients with TLE was specific to the right DLPFC and was not present at baseline. This asymmetry might be associated with the right cerebral hemisphere lateralization of cognitive networks, as mentioned in the literature (59, 60). Nevertheless, the specific mechanism and explicit implications of this finding remain to be investigated in future studies.

This study has several limitations that must not be overlooked. First, due to the lack of follow-up data for NCs, the possibility of age-related changes in the patients cannot be excluded. However, this parameter has been contained as a nuisance covariate in data analyses. Second, because the sample size of our study was somewhat small, we should replicate the current findings to elucidate DLPFC function and cognitive dysfunction in TLE with a larger sample. Third, we could not perform structural examination or analysis of the DLPFC; although considerable evidence suggests that abnormal DLPFC structure is not significantly correlated with alertness or executive dysfunction, structural assessment should nonetheless be included in future studies. Finally, as for the length of acquisition, we have other acquisitions, except the resting state fMRI, including DTI, 3D and so on. The entire acquisition lasted 35 min and the patients cannot stand for too long. Given that so many acquisitions were included, the patients were completely concentrated on the acquisitions for 6 min. We will divide more subgroups in a future study to obtain more time for acquisition.

## Conclusion

To the best of our knowledge, this is the first study to investigate alterations in the FC of the DLPFC and to examine cognitive function with the ANT in TLE patients over time. TLE patients show cognitive impairment compared to NCs as well as cognitive decline compared to their own baseline measures. In addition, aberrant FC in the DLPFC was prominent in TLE and was associated with cognitive dysfunction at the 3 year follow-up. Our new findings supplement the literature on DLPFC dysfunction and may facilitate future research on neuroimaging markers of cognitive deficits.

## Data Availability Statement

The data used in this study will be shared on request to the corresponding author.

## Ethics Statement

The studies involving human participants were reviewed and approved by the Ethics Committee of the First Affiliated Hospital of Guangxi Medical University, Nanning, China. All of the subjects were required to sign informed consent and instructed in detail about the experiment.

## Author Contributions

LQ contributed to the concept and design of this study, participated in data acquisition, analysis, interpretation of data, and the drafting of the manuscript. WJ contributed to interpretation of data and participated in drafting the manuscript. XZ and ZZ contributed to data acquisition and analysis. JL performed the statistical analysis and participated in the interpretation of data. All authors contributed to the article and approved the submitted version.

## Conflict of Interest

The authors declare that the research was conducted in the absence of any commercial or financial relationships that could be construed as a potential conflict of interest.
