# Erratum: Alterations Functional Connectivity in Temporal Lobe Epilepsy and Their Relationships With Cognitive Function: A Longitudinal Resting-State fMRI Study

**DOI:** 10.3389/fneur.2020.614081

**Published:** 2020-10-27

**Authors:** Frontiers Production Office

**Affiliations:** Frontiers Media SA, Lausanne, Switzerland

**Keywords:** temporal lobe epilepsy, functional magnetic resonance imaging, dorsolateral prefrontal cortex, longitudinal study, cognitive function

Due to a production error, incorrect affiliations were provided for Lu Qin and Wenyu Jiang. Instead of “Department of Neurology, The First Affiliated Hospital of Guangxi Medical University, Nanning, China,” it should be “Department of Neurology, Liuzhou Workers' Hospital/The Fourth Affiliated Hospital of Guangxi Medical University, Liuzhou, China” for Lu Qin, and “Department of Neurology, Jiangbin Hospital of Guangxi Zhuang Autonomous Region, Nanning, China” for Wenyu Jiang.

The publisher apologizes for this mistake. The original article has been updated.

